# Prevention of lipopolysaccharide-induced CD11b^+^ immune cell infiltration in the kidney: role of AT_2_ receptors

**DOI:** 10.1042/BSR20190429

**Published:** 2019-05-24

**Authors:** Sanket Patel, Isha Dhande, Elizabeth Alana Gray, Quaisar Ali, Tahir Hussain

**Affiliations:** Heart and Kidney Institute, Department of Pharmacological and Pharmaceutical Sciences, College of Pharmacy, University of Houston, Houston, TX 77204, U.S.A.

**Keywords:** angiotensin-II type 2 receptor, interleukin-10, lipopolysaccharide, reno-protection

## Abstract

Immune cell infiltration plays a central role in mediating endotoxemic acute kidney injury (AKI). Recently, we have reported the anti-inflammatory and reno-protective role of angiotensin-II type-2 receptor (AT_2_R) activation under chronic low-grade inflammatory condition in the obese Zucker rat model. However, the role of AT_2_R activation in preventing lipopolysaccharide (LPS)-induced early infiltration of immune cells, inflammation and AKI is not known. Mice were treated with AT_2_R agonist C21 (0.3 mg/kg), with and without AT_2_R antagonist PD123319 (5 mg/kg) prior to or concurrently with LPS (5 mg/kg) challenge. Prior-treatment with C21, but not concurrent treatment, significantly prevented the LPS-induced renal infiltration of CD11b^+^ immune cells, increase in the levels of circulating and/or renal chemotactic cytokines, particularly interleukin-6 (IL-6) and monocyte chemoattractant protein-1 (MCP-1) and markers of renal dysfunction (blood urea nitrogen and albuminuria), while preserving anti-inflammatory interleukin-10 (IL-10) production. Moreover, C21 treatment in the absence of LPS increased renal and circulating IL-10 levels. To investigate the role of IL-10 in a cross-talk between epithelial cells and monocytes, we performed *in vitro* conditioned media (CM) studies in human kidney proximal tubular epithelial (HK-2) cells and macrophages (differentiated human monocytes, THP-1 cells). These studies revealed that the conditioned-media derived from the C21-treated HK-2 cells reduced LPS-induced THP-1 tumor necrosis factor-α (TNF-α) production via IL-10 originating from HK-2 cells. Our findings suggest that prior activation of AT_2_R is prophylactic in preventing LPS-induced renal immune cell infiltration and dysfunction, possibly via IL-10 pathway.

## Introduction

Acute kidney injury (AKI) is associated with significant morbidity and mortality particularly in critically ill patients and in those who have undergone major surgery (30–60%) [1]. Multiple reports suggest that rolling and infiltration of circulating immune cells, particularly of leukocytes (monocytes and neutrophils) and natural killer T lymphocytes, is a key initiating event in the pathogenesis of AKI [2]. The homing of these cells is heterogeneous and believed to be regulated in response to local release of cytokines such as monocyte chemoattractant protein-1 (MCP-1), interleukin-6 (IL-6), tumor necrosis factor-α (TNF-α) or interleukin-10 (IL-10) by both immune cells and injured renal cells. Lipopolysaccharide (LPS), found in the outer membrane of Gram-negative bacteria, induces both strong systemic and local inflammatory responses and causes local infiltration of immune cells. The early phase of LPS-induced renal injury is characterized by persistent renal hypoperfusion (>6 h) and concomitant accumulation of waste products such as blood urea nitrogen (BUN) or creatinine or both which is highly relevant to AKI. It has been reported that depletion of mononuclear phagocytes, including monocytes, before the occurrence of kidney injury attenuated the rise in BUN and provided renoprotection [3].

The renin–angiotensin system (RAS) is a critical regulator of kidney function and influences renal inflammation and structural integrity of the kidney [4–6]. Until recently, most of the beneficial effects associated with interference of RAS function were attributed primarily to reducing angiotensin-II type 1 receptor (AT_1_R) activation. However, numerous studies suggest that angiotensin-II type 2 receptor (AT_2_R) also plays an important role in renal physiology by counteracting AT_1_R-mediated functions [7,8]. AT_2_R activation regulates both, natriuresis and the control of blood pressure in various animal models of hypertension [9–12]. The influence of AT_2_R appears to extend beyond the traditional roles of the RAS in sodium and blood pressure regulation to include anti-inflammatory actions in the kidneys [7,13–15]. Recently, using the pre-hypertensive obese Zucker rat model of chronic low-grade inflammation, we reported that 2-week treatment with the AT_2_R agonist, C21, lowered plasma and renal levels of the pro-inflammatory cytokines and reduced mesangial matrix expansion. This effect was blocked by the AT_2_R antagonist, PD123319 (PD) [7]. Moreover, AT_2_R mediated anti-inflammatory and renoprotective effects are independent of changes in blood pressure [7,16,17].

Recent *in vitro* studies indicate that AT_2_R expressed in kidney cells and phagocytes may be linked to the anti-inflammatory effects. Our prior work has demonstrated that prior-treatment with C21 lowers the production of IL-6 and TNF-α, but increases that of anti-inflammatory IL-10 in response to LPS challenge in human kidney proximal tubular epithelial (HK-2) cells [7] and THP-1 monocytes [18]. Moreover, activation of AT_2_R alone, without LPS, can stimulate IL-10 production in HK-2 cells. However in THP-1 cells, LPS was required for AT_2_R-driven increase in IL-10 levels [7]. Moreover, other *in vitro* reports suggest that AT_2_R activation concurrently or post-LPS administration does not stimulate IL-10 in THP-1 monocytes [19,20]. Prior-treatment with IL-10 has earlier been reported to modulate the release of chemotactic cytokines in humans [21] and rodents [22]. Collectively, the present study was designed to test whether prior activation of AT_2_R rather than concurrent activation protects against LPS-driven renal infiltration of immune cells and AKI potentially via the IL-10 pathway.

## Methods

### Animals

Ninety C57BL/6NHsd male mice at 8–10 weeks of age were purchased from Harlan Laboratories (Madison, WI). The animals were housed and acclimatized on light/dark cycle (lights on 7 a.m. to 7 p.m.) and 71–72°F for 1 week in the University of Houston animal care facility and had free access to standard chow and tap water. The experimental protocol was approved by the Institutional Animal Care and Use Committee.

### LPS-induced AKI protocol

The present study is divided into three protocols. All drugs administrations were intraperitoneal as we [7] and others [23] have reported and included six mice per group.

#### Protocol 1

Fifty-four mice were randomized to prior-treatment or concurrent treatment groups to study anti-inflammatory and renoprotective effects of AT_2_R activation against LPS-induced AKI 24-h post-LPS. Prior-treatment protocol: the AT_2_R agonist C21 and antagonist PD123139 alone or together were administered on days 1 (first dose) and 2 (second dose). One hour after dose 2, LPS was administered. Various groups of mice were as follows: (i) sterile saline (control), (ii) LPS (5 mg/kg), (iii) AT_2_R agonist C21 (0.3 mg/kg), (iv) C21+LPS, (v) AT_2_R antagonist PD (5 mg/kg), (vi) PD+LPS, (vii) PD+C21+LPS. Concurrent treatment protocol: C21 alone or with PD123319 were given once 1 h prior to LPS administration. Treatment groups are as follows: (viii) C21+LPS (ix) PD+C21+LPS. All groups of mice 24-h post-LPS were killed.

#### Protocol 2

Another 24 mice were randomly assigned to prior-treatment groups to study the immediate effects of AT_2_R activation on LPS-induced inflammatory response 2-h post-LPS. In this protocol we used the following group of mice: (i) sterile saline (control), (ii) LPS (5 mg/kg), (iii) AT_2_R agonist C21 (0.3 mg/kg) or (iv) C21+LPS. As aforementioned, the prior-treatment group included C21 administration of two doses on days 1 and 2. One hour after the second dose of C21, LPS was administered and then 2 h later, the mice were killed.

#### Protocol 3

Another 12 mice were randomized to receive (i) sterile saline or (ii) AT_2_R agonist C21 (0.3 mg/kg) on days 1 (first dose) and 2 (second dose) to determine the immediate effects of AT_2_R activation on circulating and renal IL-10 at the time of LPS was given in above protocols. These mice were not subjected to LPS challenge. The mice were killed 1 h after the second dose of C21.

All mice were euthanized under isoflurane anesthesia. Spot urine was collected from the bladder. Biochemical analysis performed in spot/random urine sample was correlated with urine sample collected over 24 h from experimental animals [24,25] or from patients [26–28]. Blood for plasma was collected in Vacuette® K_3_-EDTA-coated tubes (95057-227, VWR, Radnor, PA) through cardiac puncture and kidneys were harvested and stored at −80°C. Plasma was prepared by centrifugation (700×***g***, 20 min and 4°C) and stored at −80°C. In three mice from each group (protocol 1 only), the right kidney was clamped before whole-body fixation with sterile 4% sucrose in phosphate buffered saline (PBS) followed by formalin-free fixative (Milestone Medical). Kidneys were collected and kept in fixative for 24 h at 4°C and transferred to 30% sucrose in PBS for preparation of cryopreservation. Tissues were embedded in Optimal Cutting Temperature compound (OCT) and preserved at −80°C for microscopy.

### Direct immunolabeling and confocal microscopy

The OCT-embedded kidney blocks were maintained at −23°C in a cryotome and three 15–30 μ thick sections per block were retrieved on ethylene glycol-based cryoprotectant. Kidney sections were washed (4 × 10 min) using PBS-(0.5%) tween-20 (PBST) and incubated with blocking buffer (PBST containing normal donkey serum (10%, 102643-998, Jackson Immunoresearch Laboratories), bovine serum albumin (1%, 101175-892, Thermo Fisher Scientific) and saponin (0.05%, 47036, Sigma–Aldrich)) for 1 h at room temperature. Kidney sections were directly immunolabeled with Alexa Fluor® 594 anti-mouse/human CD11b (1:400, 101254, Biolegend) overnight at 4°C. Sections were washed (4 × 10 min) using PBST and counterstained with DAPI (3 μM, D1306, Thermo Fisher Scientific). Sections were mounted on slide using FluoroGel mounting media (17985-02, Electron Microscopy Sciences), sealed with clear nail polish and analyzed on Leica SP8 Confocal Microscope (63×/1.4 N.A. oil objective lens, 1 AU, 0.75× zoom, 0.3 μ Z-step, 2–4% laser power).

### Cytokine measurements by ELISA

Cytokines IL-6, IL-10 and TNF-α in the plasma and renal cortex homogenates were determined by Mouse Quantikine ELISA kit M1000B, M6000B and MTA00B (R&D Systems Inc.), respectively, as we have described earlier [7,18]. MCP-1 was measured by mouse ELISA kit (BMS6005, Thermo Scientific).

### Measurement of plasma and urinary markers of renal function

Plasma and urinary biomarkers of renal function such as BUN (QuantiChrome urea assay kit, DIUR-100), and creatinine content (QuantiChrome creatinine assay kit, DICT-500) were measured spectrophotometrically according to manufacturer’s instructions (BioAssay Systems). Albuminuria was determined spectrophotometrically using ELISA kit (E99-134, Bethyl Laboratories, Inc.).

### Conditioned media experiments

#### Preparation of conditioned medium

Conditioned medium (CM) from HK-2 cells was used to determine the effect of AT_2_R agonist treated proximal tubular epithelial cells (PTECs) on LPS-activation of THP-1 macrophage. Control CM (CCM, vehicle treatment) and CM (C21-treated HK-2 cells) was prepared based on the method described by Wang et al. [29]. Confluent HK-2 cells in 100-mm culture plates were washed twice with K-SFM and then incubated in 5 ml of K-SFM with bovine pituitary extract and epidermal growth factor alone or 5 ml of medium containing 1 µmol/l C21 for 24 h at 37°C. At the end of 24 h, the media were removed, washed twice with K-SFM Ham, replaced with fresh medium and incubated for a further 24 h. Media were then collected, filtered through a 0.2-µm filter and used immediately for treatment in THP-1 macrophages. Medium incubated with C21 was designated CM and medium without C21 was designated as CCM. Two milliliters of aliquots of CM were taken to generate IL-10-free CM as described below.

#### Preparation of IL-10-free CM

A portion of the CM was made IL-10-free (IL-10-free CM) utilizing IL-10 neutralizing antibody as described by Endharti et al. [30]. CM was incubated with 1 μg/ml anti-IL-10 antibody by gentle mixing for 2 h. Protein G resin was added to the Ag–Ab complex (50 μl of resin per 10 μg of Ab), and samples were incubated with gentle mixing overnight at 4°C. Immobilized protein G-bound complexes were removed from the CM by centrifugation at 2500×***g*** for 5 min. Supernatant was designated as IL-10-free CM. To ascertain the amount of IL-10 cytokine expression in the media, IL-10 was measured using ELISA.

#### Treatment of THP-1 macrophages with CM from HK-2 cells

**Figure 1:**
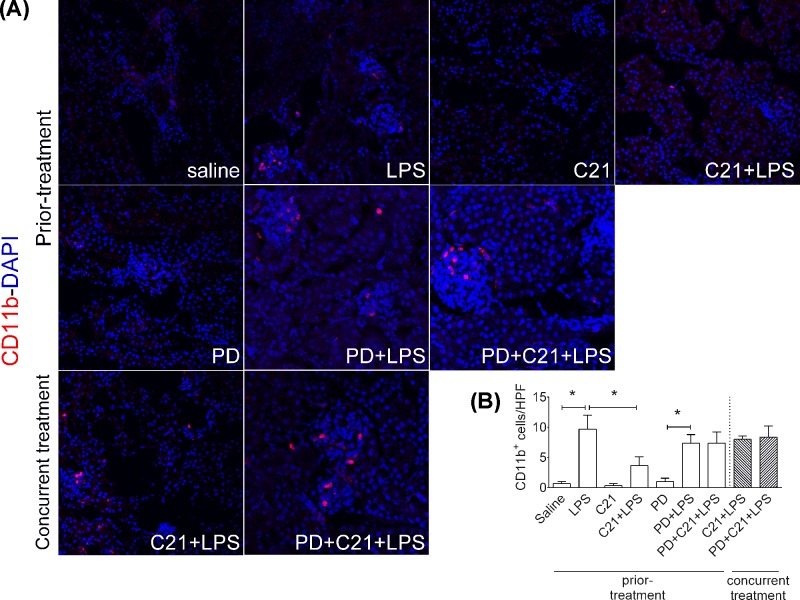
AT_2_R agonist C21 prevents lipopolysaccharide-induced renal infiltration of CD11b+ immune cells in mice Direct immunolabeling of CD11b^+^ immune cells in kidney (**A**) 24-h post-LPS. Mice were treated prior or concurrently with sterile saline, AT_2_R agonist C21 (0.3 mg/kg) or antagonist PD123319 (5 mg/kg) or LPS (5 mg/kg) intraperitoneally as explained in Protocol 1. Fine-fixed™ and free-floating kidney sections (15–30 μ) were washed, permeabilized, stained with Alexa Fluor-594 conjugated mouse specific anti-CD11b antibody and DAPI nuclear stain and mounted on a glass microscope slide. Images were acquired and analyzed on Leica SP8 Confocal Microscope (63×/1.4 N.A. oil objective lens, 1 AU, 0.75× zoom, 0.3 μ Z-step, 2–4% laser power). The CD11b^+^ immune cells per high power field were manually counted (246 μ × 246 μ) and analyzed as an index of immune cell infiltration (**B**). At least, three images per section, three sections per mouse kidney and three mouse kidneys per group were analyzed. Number of CD11b^+^ cells per nine images per mice was averaged and mean ± S.E.M. of three mice per group was presented. Data were analyzed by one-way ANOVA with Fisher’s LSD test for multiple comparisons and considered significant at *P<0.05. Note: *concurrent treatment groups are shown with hatched bars.* The graph bars which mark no symbol means they are not statistically significant.

THP-1 macrophages were derived from monocytes by differentiating with phorbol 12-myristate, as we described earlier [18]. After the resting phase, CCM, CM or IL-10-free CM was added to macrophages 1 h prior to activation with LPS (1 µg/ml) (Figure 1). Medium was collected 24-h post-LPS, cleared via 0.2-μm syringe filter, and TNF-α and IL-10 were quantitated by ELISA.

### Statistical analyses

Data are presented as mean ± S.E.M. One-way or two-way ANOVA with Fisher’s LSD test for multiple comparisons was used to compare variations between more than two groups. A value of *P*<0.05 was considered statistically significant, *n*=6 for the *in vivo* treatments and with *n*=5–8 per group for the *in vitro* experiments. The concurrent treatment groups are *italicized* and underlined in-text and shown with *hatched* bars in-figure to differentiate from the prior-treatment groups.

## Results

### Infiltration of CD11b^+^ immune cells

Leukocytes (monocytes and neutrophils) and natural killer T lymphocytes stably express cell surface protein CD11b. Staining of tissue sections for CD11b convincingly demonstrates the degree of their infiltration. Thick free-floating kidney sections were stained for CD11b^+^ (Figure 1A) and increase in number of CD11b^+^ cells was considered as an index of immune cell infiltration (Figure 1B, all values are reported in number of cells per high power field (HPF)). LPS administration caused significant renal infiltration of CD11b^+^ immune cells as compared with saline controls (LPS: 9.7 ± 2.3 vs. saline: 0.7 ± 0.3). Prior-treatment with C21 only was able to reduce renal immune cell infiltration under endotoxemia (C21+LPS: 3.7 ± 1.5). The AT_2_R antagonist PD reversed C21-mediated reduction in immune cell infiltration (PD+C21+LPS: 7.3 ± 1.5). Concurrent C21 (without or with PD) treatment with LPS did not affect LPS-induced renal infiltration of immune cells (*C21*+*LPS*: 8.0 ± 0.6, *PD*+*C21*+*LPS*: 8.3 ± 1.9). The AT_2_R agonist C21 or the antagonist PD in absence of LPS did not affect immune cell infiltration (C21: 0.3 ± 0.3, PD: 1.0 ± 0.6). Also, the AT_2_R antagonist PD had no significant effect on LPS-induced immune cell infiltration (PD+LPS: 7.3 ± 1.5 vs LPS: 9.7 ± 2.3).

### Renal dysfunction

**Figure 2:**
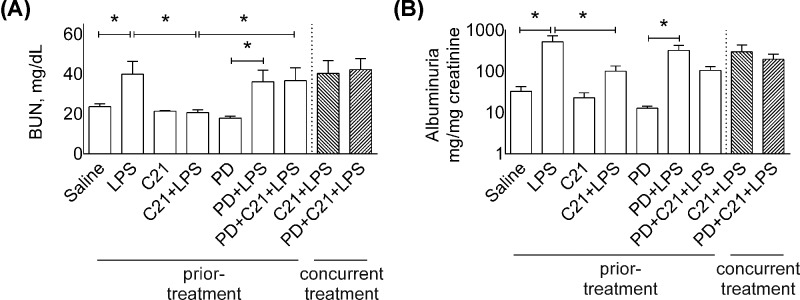
Effect of AT_2_R agonist C21 on indices of renal dysfunction in mice BUN (**A**) and albuminuria (urinary albumin-to-creatinine ratio, mg/mg) (**B**), the biomarkers of AKI 24-h post-LPS. Mice were treated prior or concurrently with sterile saline, AT_2_R agonist C21 (0.3 mg/kg) or antagonist PD123319 (5 mg/kg) or LPS (5 mg/kg) intraperitoneally as explained in Protocol 1. Data are represented as mean ± S.E.M., analyzed by one-way ANOVA with Fisher’s LSD test for multiple comparisons and are considered significant at *P<0.05; *n*=6 per group. Note: *concurrent treatment groups are shown with hatched bars.* The graph bars which mark no symbol means they are not statistically significant.

BUN and albuminuria (urinary albumin-to-creatinine ratio) as the classical markers of AKI were measured 24 h after LPS challenge. LPS treatment caused a significant increase in BUN (LPS: 40 ± 6 vs. saline: 24 ± 1) (Figure 2A, all mg/dl) and albuminuria (LPS: 519 ± 211 vs. saline: 33 ± 10) (Figure 2B), which were ameliorated by C21 prior-treatment (BUN- C21+LPS: 20 ± 1; albuminuria- C21+LPS: 100 ± 35). The effect of C21 on BUN was attenuated by the AT_2_R antagonist PD (PD+C21+LPS: 37 ± 6), but albuminuria remained unaffected (PD+C21+LPS: 106 ± 24). Concurrent treatment with C21 had no effects on LPS-induced BUN and albuminuria (*C21*+*LPS*: 40 ± 6 BUN; 298 ± 136 albuminuria) compared with LPS alone.

### Plasma pro- and anti-inflammatory cytokines

**Figure 3:**
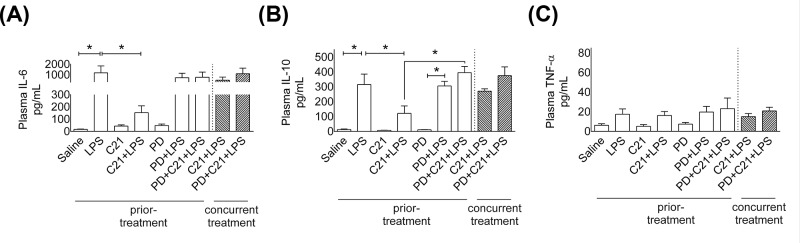
Effect of AT_2_R agonist C21 on levels of plamsa cytokines 24-hr post-LPS in mice Levels of IL-6 (**A**), IL-10 (**B**) and TNF-α (**C**) in plasma 24-h post-LPS. Mice were treated prior or concurrently with sterile saline, AT_2_R agonist C21 (0.3 mg/kg) or antagonist PD123319 (5 mg/kg) or LPS (5 mg/kg) intraperitoneally as explained in Protocol 1. Data are represented as mean ± S.E.M., analyzed by one-way ANOVA with Fisher’s LSD test for multiple comparisons and are considered significant at *P<0.05; *n*=6 per group. Note: *concurrent treatment groups are shown with hatched bars.* The graph bars which mark no symbol means they are not statistically significant.

Plasma was collected at 24-h post-LPS to evaluate the inflammatory response (all pg/ml plasma). LPS treatment resulted in an increase in IL-6 (LPS: 1183 ± 650 vs. saline: 16 ± 3) (Figure 3A) and IL-10 (LPS: 315 ± 70 vs. saline: 12 ± 4) (Figure 3B). The increase in LPS-induced IL-6 and IL-10 was attenuated by C21 prior-treatment (C21+LPS: 152 ± 55 (IL-6), 121 ± 50 (IL-10)) as compared with LPS treated mice. The AT_2_R antagonist PD blocked C21-mediated reduction in circulating IL-10, but not IL-6. Consistent with the results of immune cell infiltration and renal dysfunction, concurrent treatment with C21 did not affect LPS-induced IL-6 or IL-10. Interestingly, in 24-h post-LPS plasma, unlike IL-6 and IL-10 changes, TNF-α levels either by LPS or the other drug treatments remained unchanged (Figure 3C).

**Figure 4:**
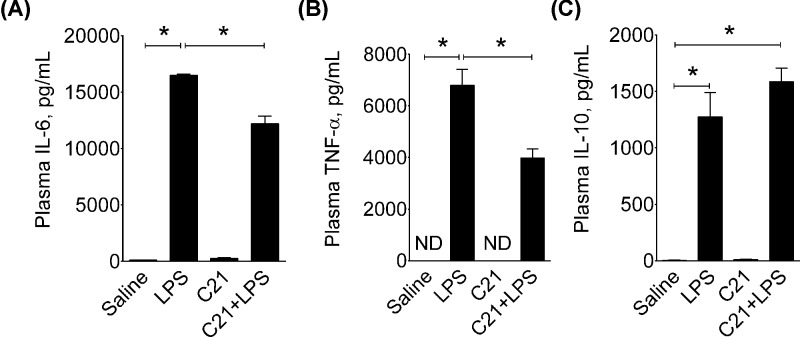
Effect of AT_2_R agonist C21 on levels of plasma cytokines 2-hr post-LPS in mice Levels of IL-6 (**A**), TNF-α (**B**) and IL-10 (**C**) in the plasma 2-h post-LPS. Mice were treated intraperitoneally with sterile saline, AT_2_R agonist C21 (0.3 mg/kg) or LPS (5 mg/kg) as explained in Protocol 2. Data are represented as mean ± S.E.M., analyzed by one-way ANOVA with Fisher’s LSD test for multiple comparisons and are considered significant at *P<0.05; *n*=6 per group. The graph bars which mark no symbol means they are not statistically significant.

In another set of animals, plasma was also collected at 2-h post-LPS to determine the early inflammatory response to LPS and/or AT_2_R agonist C21. LPS treatment not only caused an increase in IL-6 (LPS: 16498 ± 113 vs. saline: 102 ± 15) (Figure 4A), TNF-α (LPS: 6798 ± 612 vs. saline: non-detectable) (Figure 4B) but also caused a profound increase in IL-10 (LPS: 1274 ± 217 vs. saline: 4 ± 1) within 2 h (Figure 4C). In mice treated with C21 prior to LPS, the levels of IL-6 (C21+LPS: 12210 ± 681) and TNF-α were significantly reduced (C21+LPS: 3986 ± 342), while IL-10 levels remained modestly, but non-significantly, high (∼25%) (C21+LPS: 1586 ± 119) as compared with the LPS group. Levels of these cytokines in saline treated controls were very low or non-detectable and were not altered by C21 treatment alone.

### Renal pro- and anti-inflammatory cytokines

**Figure 5:**
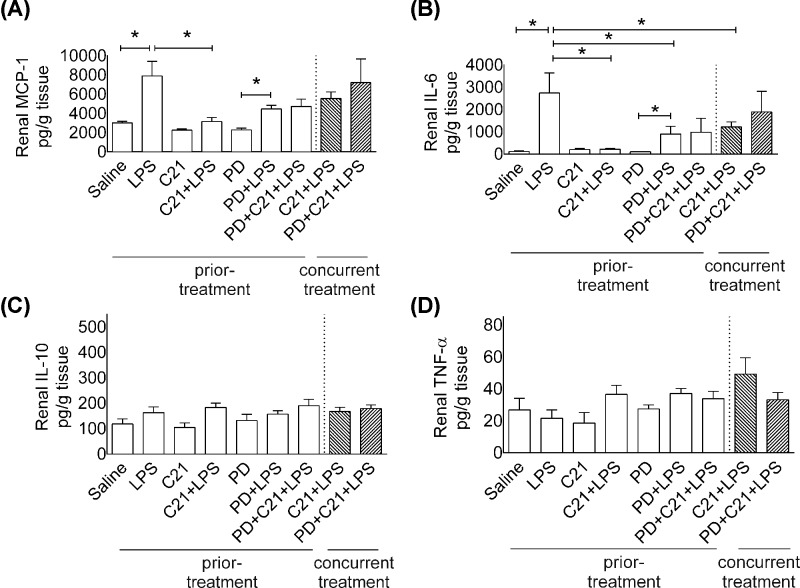
Effect of AT_2_R agonist C21 on levels of renal cytokines 24-hr post-LPS in mice Levels of MCP-1 (**A**), IL-6 (**B**), IL-10 (**C**) and TNF-α (**D**) in kidney 24-h post-LPS. Mice were treated prior or concurrently with sterile saline, AT_2_R agonist C21 (0.3 mg/kg) or antagonist PD123319 (5 mg/kg) or LPS (5 mg/kg) intraperitoneally as explained in Protocol 1. Data are represented as mean ± S.E.M., analyzed by one-way ANOVA with Fisher’s LSD test for multiple comparisons and are considered significant at *P<0.05; *n*=6 per group. Note: *concurrent treatment groups are shown with hatched bars.* The graph bars which mark no symbol means they are not statistically significant.

Kidneys were harvested at 24-h post-LPS treatment to quantitate cytokines in the cortical homogenates. LPS treatment significantly increased (all pg/g kidney) MCP-1 (LPS: 7892 ± 1498 vs. saline: 2998 ± 180) (Figure 5A), IL-6 (LPS: 2735 ± 899 vs. saline: 113 ± 26) (Figure 5B), while IL-10 (Figure 5C) and TNF-α (Figure 5D) were relatively low and remained unchanged in all treatment groups at 24 h. Treatment with C21 prior to LPS administration completely prevented this increase in renal MCP-1 (C21+LPS: 3136 ± 423) and IL-6 (C21+LPS: 216 ± 33). PD treatment produced modest but not significant attenuation of the C21-medited reduction in MCP-1 and IL-6 (PD+C21+LPS: 4717 ± 752 MCP-1; 979 ± 621 IL-6). Concurrent treatment with C21 or PD did not affect MCP-1 (*C21*+*LPS*: 5548 ± 675; *PD*+*C21*+*LPS*: 7196 ± 2449) and IL-6 (*C21*+*LPS*: 1218 ± 220; *PD*+*C21*+*LPS*: 1888 ± 918).

### Effect of AT_2_R agonist C21 on plasma and renal IL-10 at time of LPS challenge

**Figure 6:**
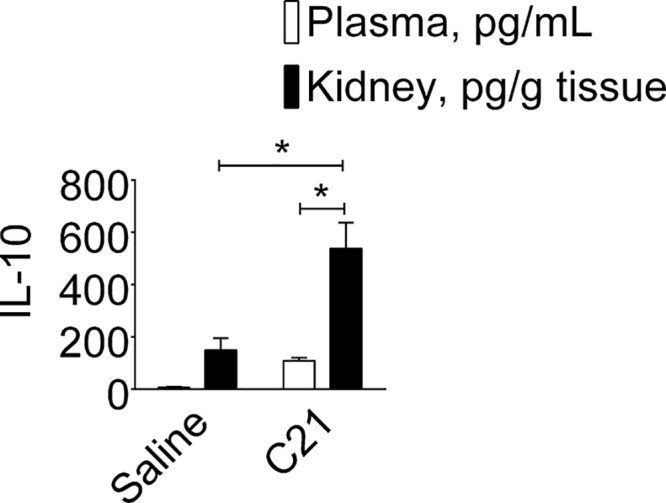
Effect of AT_2_R activation on IL-10 in the plasma (pg/mL plasma) and the kidney (pg/g kidney) in absence of LPS Mice were treated intraperitoneally with sterile saline or AT_2_R agonist C21 (0.3 mg/kg) as explained in Protocol 3. Data are represented as mean ± S.E.M., analyzed by two-way ANOVA with Fisher’s LSD test for multiple comparisons and are considered significant at *P<0.05; *n*=6 per group. The graph bars which marks no symbol means they are not statistically significant.

In another group of control and C21 treated mice, IL-10 was measured in the plasma and kidney 1 h after the second dose of C21 to assess the levels of IL-10 prior to LPS administration. Levels of anti-inflammatory cytokine IL-10 were only modestly (non-significant) increased in plasma (C21: 108 ± 11 vs. saline: 6 ± 3 pg/ml) but significantly increased in kidney (C21: 538 ± 99 vs. saline: 149 ± 45 pg/g kidney) of AT_2_R agonist C21 treated mice (Figure 6). However, equi-volume comparisons, that is, per milliliter plasma vs per gram kidney, show that renal IL-10 levels were several fold higher than the plasma in response to C21.

**Figure 7:**
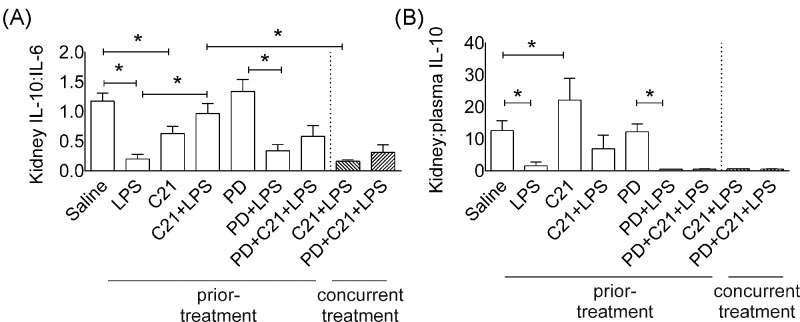
Indirect assessment of effect of AT_2_R agonist on kidney IL-10 relative to kidney IL-6 and plasma IL-10 Effect of AT_2_R activation on protein expression of anti-inflammatory IL-10 in the kidney relative to IL-6 (Kidney IL-10:IL-6) (**A**) and plasma IL-10 (kidney-to-plasma IL-10) (**B**) as indirect assessment of renal microenvironment 24-hr post-LPS. Data are represented as mean ± S.E.M., analyzed by one-way ANOVA with Fisher’s LSD test for multiple comparisons and are considered significant at *P<0.05; *n*=6 per group. Note: concurrent treatment groups are shown with hatched bars. The graph bars which marks no symbol means they are not statistically significant.

Inflammation and time-course of cytokines available in circulation and in renal microenvironment greatly varies. Thus, renal recruitment of immune cells is primarily dependent on balance among inflammatory and anti-inflammatory cytokines. The AT_2_R and LPS, have been shown to activate renal epithelial and immune cells and the release of cytokines, IL-6 and IL-10 [7,18]. Hence, we have indirectly estimated local (kidney) IL-10 content relative to kidney IL-6 (kidney IL-10:IL-6) (Figure 7A) and plasma IL-10 (kidney-to-plasma IL-10) (Figure 7B) to assess the effect of AT_2_R activation on IL-10 24-h post-LPS to correlate with results showing kidney dysfunction. The effects of LPS and C21 were distinct; LPS and C21, both significantly depressed kidney IL-10:IL-6 ratio, but prior-treatment of C21 improved kidney IL-10:IL-6 ratio. Concurrent treatment with C21 did not affect kidney IL-10:IL-6 ratio. PD treatment did not significantly block effect of C21 on kidney IL-10:IL-6 (Figure 7A). Likewise, LPS significantly reduced kidney:plasma IL-10 that was modestly improved with C21 prior-treatment, but not with concurrent treatment (Figure 7B). C21 treatment alone in absence of LPS also increased kidney:plasma IL-10. Nonetheless, the kidney-to-plasma IL-10 and kidney IL-10:IL-6 ratios remained higher in C21+LPS mice as compared with LPS mice or mice concurrently treated with C21, PD or LPS (Figure 7A,B).

### Effect of AT_2_R agonist C21 treated HK-2 cell-derived CM on LPS-induced cytokine production in THP-1 macrophages

**Figure 8:**
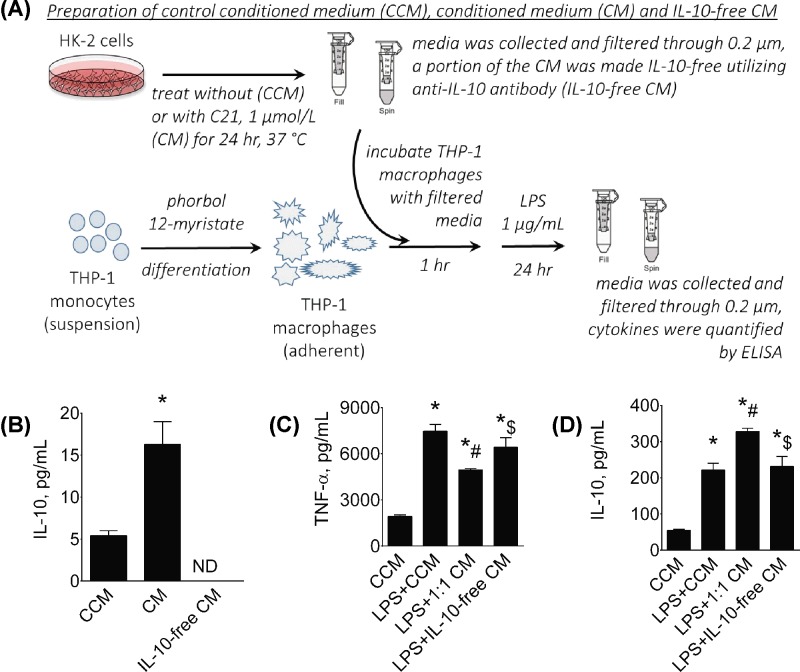
Conditioned media experiment Schematic representation of conditioned media experiment (**A**), effect of control conditioned media (CCM), conditioned media (CM), and IL-10-free CM derived from HK-2 cells stimulated with AT_2_R agonist C21 (1 µmol/L) on release of IL-10 from THP-1 macrophages (**B**). Effect of prior-treatment of CCM, CM, and IL-10-free CM derived from HK-2 cells on release of TNF-α (**C**) and IL-10 (**D**) from THP-1 macrophages stimulated with LPS. THP-1 macrophages were treated with CCM, CM or IL-10-free CM 1-hour prior to activation with LPS (1 μg/mL). Media was collected 24-hour post-LPS and TNF-α and IL-10 were quantified by ELISA. Data are represented as mean ± S.E.M., analyzed by one-way ANOVA with Fisher’s LSD test for multiple comparisons and are considered significant at *P*<0.05 *vs. CCM, #vs. LPS+CCM; $vs. LPS+1:1 CM; *n*=5-8.

Schematic representation of conditioned media experiment is presented (Figure 8A). CM from C21-treated HK-2 cells was used to determine whether proximal tubular epithelial HK-2 cells treated with AT_2_R agonist could inhibit LPS-induced activation of macrophage. CM, CCM and IL-10-free CM were prepared as described in the ‘Methods’ section and IL-10 produced in each type of CM was determined by ELISA (all pg/ml). IL-10 concentration in CM was three-fold higher compared with CCM (CM: 16 ± 3 vs. CCM: 5 ± 1) and IL-10-free CM contained non-detectable amounts of IL-10 (Figure 8B). Treatment with CM from C21-treated HK-2 cells resulted in a ∼33% decrease in LPS-induced TNF-α production (LPS+1:1CM: 4952 ± 72 vs. LPS+CCM: 7465 ± 438) (Figure 8C) but a ∼30% increase in IL-10 production (LPS+1:1CM: 328 ± 9 vs. LPS+CCM: 221 ± 19) (Figure 8D) in THP-1 macrophages. However, treatment with IL-10-free CM abrogated the anti-inflammatory effect of CM from C21-treated HK-2 cells and the cytokine levels were comparable with those obtained with LPS+CCM treatment (LPS+IL-10-free CM: 6421 ± 618 (TNF-α), 231 ± 28 (IL-10)).

## Discussion

Activation, tethering and rolling of circulatory immune cells, primarily leukocytes (neutrophils and monocytes) and lymphocytes and their subsequent tissue extravasation is one of the common features during inflammatory injury [31]. Under inflammatory stimulus such as LPS, immune cells rapidly infiltrate kidney and drive injury [2]. Here, we attempted to identify the involvement of the AT_2_R in preventing renal accumulation of CD11b^+^ immune cells that mainly include monocytes, neutrophils and natural killer cells, potential LPS-driven mediators of renal inflammation and injury. And our findings clearly demonstrate that prior, but not concurrent stimulation of AT_2_R is essential to suppress renal infiltration of CD11b^+^ cells. In general, most of the beneficial C21-mediated effects were reversed by the AT_2_R antagonist PD, suggesting involvement of AT_2_R in renoprotection. In fact, acute treatment of PD did not exacerbate renal injury upon LPS challenge. But, chronic treatment of obese rats with PD showed increase in renal mesangial matrix expansion, increased renal IL-6 but reduced renal IL-10 content [7]. Collectively, it may be inferred that AT_2_R have intrinsic tone in renoprotection in the long term. A prophylactic approach using prior-treatment to mitigate renal inflammation has been shown to exert better outcomes [32–35]. If immune cell infiltration remains unresolved (i.e. in case of LPS or *C21+LPS* concurrent group), monocytes eventually differentiate within 48–72 h post-LPS into either pro-inflammatory M1-type macrophage and/or phenotypically distinguished dendritic cells and propagate renal injury. Alternatively, they may initiate tissue repair upon maturation into anti-inflammatory M2-type macrophage phenotype. It infers that 24 h is not sufficient to study differentiation of such cells, thus in present study we did not characterize CD11b^+^ infiltrated cells further. We have previously shown that chronic activation of AT_2_R reduced renal infiltration of macrophages in obese Zucker rat, an animal model characterized by low-grade chronic systemic inflammation [7].

Decreased vascular resistance, arterial vasodilation, associated renal hypoperfusion and hypotension are hallmarks of endotoxemia and considered as causes of AKI [1]. However, the dose of LPS employed in our present work does not affect blood pressure [36,37]. Renal hemodynamics may change under endotoxemia in the absence of blood pressure change. As a result of reduced renal blood flow, the nitrogenous waste products (i.e. BUN) may acutely build up in body. Literature suggests that detectable but stable rises in BUN and albuminuria symbolize AKI and they can be studied in parallel along with infiltration of immune cells at 24 h [3,34,38,39]. Thus, we determined rise in BUN and albuminuria along with infiltration of CD11b^+^ immune cells, all at 24-h post-LPS endotoxemia; which were all mitigated by C21 prior-treatment. However, the absence of reversal by PD of effect of C21 on albuminuria is not known; a shorter half-life of PD (∼20 min) can be considered a factor [40,41]. We have used low dose of C21 and therefore, whether C21 effect on albuminuria is independent of AT_2_R requires further studies. Literature reveals nothing as to the non-AT_2_R as a C21 target [42], but such possibility cannot be ruled out. In line with the above notion that signifies a causal role of reduced blood flow or renal hypoperfusion during endotoxemia-induced renal immune cell infiltration and injury, we infer that the approach improving or normalizing renal perfusion should limit incident AKI [43]. We [11] and others [12,44] have reported that selective activation of AT_2_R has improved renal hemodynamics and this may constitute one of the mechanisms by which AT_2_R agonist C21 exerts renoprotection. The blood pressure-independent anti-inflammatory effects of AT_2_R agonist has earlier been reported [7,16,17].

The temporal and spatial cytokine response to LPS is heterogeneous and greatly varies among individuals and across species [45,46] due to the involvement of multiple types of immune cells, receptors and pleiotropic effects of numerous cytokines [47,48]. Systemic acute endotoxemia in mice includes a monophasic spike in circulating TNF-α and persistent elevation of IL-6 [49] that is consistent with our present findings. Specifically, we observed that prior-treatment with AT_2_R agonist C21 reduced the immediate rise in circulatory chemotactic TNF-α and IL-6 but preserved anti-inflammatory IL-10 (∼+25%) at 2 h in response to LPS. At 24-h post-LPS challenge, the levels of TNF-α mostly remained low and IL-6 remained high. Similarly, as a late response, at 24 h after LPS administration, prior-treatment with AT_2_R agonist C21 significantly normalized MCP-1 and IL-6. MCP-1, TNF-α and IL-6 are potent drivers of immune cell infiltration in mice undergoing endotoxemia [38,50,51]. However, the relative contribution of MCP-1, IL-6 or TNF-α in LPS-induced inflammatory injury is difficult to assess. However, renal IL-10 levels did not sustain 24-h post-LPS among study groups. It is plausible that C21-induced early increase helps resolve inflammation and does not require it to sustain high at later time. We speculate that activation of AT_2_R may have an immunomodulatory effect through IL-10 that may prime renal and immune cells to fight against greater insult, i.e. LPS. Specifically, we have reported that AT_2_R agonist C21 increased IL-10 in rat kidney and human kidney cells (HK-2 cells) [7] as well as THP-1 macrophages [18]. The present study does not reveal the cell type involved in decreased plasma IL-10 levels upon prior-treatment with C21 that remains a limitation.

IL-10 is a potent anti-inflammatory cytokine which has been shown to counter-regulate TNF-α production [7,18,19], produce renal anti-fibrotic effects [52,53], and protect against renal ischemia [16]. Post-operative IL-10 content is significantly associated with lower risk of mortality [54]. Studies indicate that AT_2_R activation stimulates IL-10 expression in the kidney, including the PTECs [7], macrophages [18] and lymphocytes [55]. Our *in vitro* and *in vivo* findings imply that upon AT_2_R activation, IL-10 was sufficiently present within the renal microenvironment at time of LPS challenge that may have played a role in attenuating LPS-mediated increase in renal TNF-α and thus infiltration of immune cells and injury. *In vitro* experiment revealed that CM derived from C21-treated HK-2 cells increased IL-10 in a range of 100 pg/ml (i.e. LPS+1:1 CM vs. LPS+CCM), concentration at which IL-10 was able to reduce TNF-α. This is supported with our *in vivo* findings that AT_2_R stimulation under normal condition can elevate IL-10 in a similar manner (e.g. at 1-h post-C21, IL-10: 538 pg/g kidney, 149 pg/ml plasma) at time of LPS challenge which may have exerted renoprotection. This anti-inflammatory response was abolished when medium was neutralized with anti-IL-10 antibody. Precisely which pathways are activated by the AT_2_R agonist C21 treatment when administered prior to or post-LPS challenge requires further investigation.

It can be argued that tissues other than kidney may contribute to production of IL-10 upon C21 activation. If that is the case, we would expect these tissues to release IL-10 in circulation which is expected to increase higher plasma IL-10 content and is essential for IL-10’s paracrine effect. But in our study, plasma IL-10 remained low (vs. kidney content) upon C21 stimulation at time of LPS challenge as per Protocol 3. Even at later time-point, i.e. 24-h post-LPS, renal IL-10 content was higher as compared with plasma IL-10 (kidney-to-plasma IL-10) and kidney IL-6 content (kidney IL-10:IL-6) upon C21 prior-treatment. This strongly suggests that prior activation renal AT_2_R increases production of IL-10 in renal microenvironment that is protective. Moreover, drug that is expected to accumulate in the body may produce profound effect upon multiple daily administration. However, C21 is not expected to accumulate upon intraperitoneal administration based on its reported half-life in rat (∼4 h) [56]. Therefore, we reiterate that beneficial effects of AT_2_R agonist C21 observed herein are not due to number of doses, i.e. two (prior-treatment) vs. one (concurrent treatment).

In summary, we conclude that prior activation of AT_2_R, and not the concurrent activation, attenuates LPS-stimulated immune cell infiltration, renal dysfunction and pro-inflammatory cytokine storm, which may be linked to increased renal production of anti-inflammatory IL-10, while the systemic anti-inflammatory response may play a smaller role. It is interesting to note that prevention of CD11b^+^ cell infiltration was very much in line with the normalization of renal function by AT_2_R agonist treatment suggesting these were the major immune cells involved in LPS-induced renal injury. However, a better understanding of the protective mechanisms of renal AT_2_R activation that can lower LPS-mediated renal infiltration of heterogeneous immune cell population may further be explored in kidney-specific AT_2_R-knockout animals or by delivering AT_2_R agonist C21 directly into the kidney cortex. Nonetheless, present findings reveal AT_2_R-IL-10 pathway as a novel therapeutic strategy for the treatment of inflammatory renal injury.

## Clinical perspectives

- Immune cell infiltration is a key event in incident AKI. Our findings suggest that a prophylactic approach with selective AT_2_R agonist C21 to boost anti-inflammatory IL-10 is relevant to patients in preventing infiltration immune cells and incident AKI. In fact, incident AKI and progression of chronic kidney disease are integrally involved in a self-propagating loop (acute-on-chronic) and there is a pressing need to halt this cycle.
- Our study reveals the importance of local (renal) IL-10 production in contributing to the anti-inflammatory effects of AT_2_R agonist treatment.
- The notion that priming of kidney through AT_2_R-IL-10 is unique and may be considered by clinicians to prevent kidney injury. Along the same line the treatment with AT_2_R agonist and IL-10 antibody may improve health outcomes.

## Abbreviations

AKI: acute kidney injury
ANOVA: analysis of variance
AT_1_R: angiotensin-II type 1 receptor
AT_2_R: angiotensin-II type 2 receptor
BUN: blood urea nitrogen
CCM: control conditioned medium
CM: conditioned medium
IL-6: interleukin-6
IL-10: interleukin-10
K-SFM: keratinocyte serum-free media
LPS: lipopolysaccharide
LSD: least significant difference
MCP-1: monocyte chemoattractant protein-1
OCT: Optimal Cutting Temperature compound
PBS: phosphate buffered saline
PBST: PBS-(0.5%) tween-20
PTEC: proximal tubular epithelial cell
RAS: renin–angiotensin system
TNF-α: tumor necrosis factor-α
